# Upper‐limb acute superficial lymphatic thrombosis: A case report

**DOI:** 10.1002/ccr3.6387

**Published:** 2022-10-03

**Authors:** Rémy Hamdan, Nicolas Briche, Vanessa Gasmi, Ilham Abejiou, Ingrid Lafon, Lounes Djerroudi

**Affiliations:** ^1^ Department of Angiology Dijon Bourgogne University Hospital Dijon France; ^2^ Department of Clinical Hematology Dijon Bourgogne University Hospital Dijon France; ^3^ Department of Diagnostic and Theranostic Medicine Institute Curie Paris France

**Keywords:** acute lymphangitis, adverse effect, coagulation, lymphatic thrombosis, skin biopsy

## Abstract

Upper‐limb acute superficial lymphatic is a rare phenomenon that has received little attention in the medical literature to date, yet it mimics superficial venous thrombosis and may also complicate a skin punch biopsy.

## Graphical abstract

This report highlights the clinical implications of in vitro‐demonstrated lymphatic vessels' ability to thrombose. The differential diagnosis list for superficial venous thrombosis is enlarged. Like veins, hypercoagulability, endothelial changes, and stasis may result in acute lymphatic thrombosis. Additionally, here, we report for the first time an unknown acute skin biopsy complication.

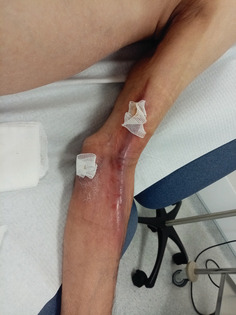

A 56‐year‐old patient was hospitalized for left‐arm cellulitis with acute lymphangitis and hyperleukocytosis of 138.9 × 10^9^/L. A bone marrow aspiration revealed acute monocytic leukemia (93% blasts). Induction chemotherapy and antibiotics were started, and the leucocyte count quickly became normal.

On the 16th day, a 6‐mm punch biopsy of erythematous plaque in the path of the lymphangitis revealed keratinocyte necrosis, a dermal non‐specific histiocyte‐rich infiltrate (Figure 1), with no evidence of leukemic infiltration. Podoplanin immunostaining revealed numerous lymphatics (Figure 2). A thick and erythematous cord extending upstream from the biopsied site occurred the following day (Figure 3). Leukocytes were 9.5 × 10^9^/L, C‐reactive protein was negative, D‐dimers were 2530 μg/L, skin and blood samples were sterile, upper‐limb Doppler ultrasound ruled out venous thrombosis and revealed a 6‐mm‐diameter incompressible non‐circulating channel; so, the diagnosis of superficial acute lymphatic thrombosis was made. The antibiotics were continued for 7 days, and enoxaparin 40 mg daily was prescribed for 6 weeks, resulting in complete remission.

**FIGURE 1:**
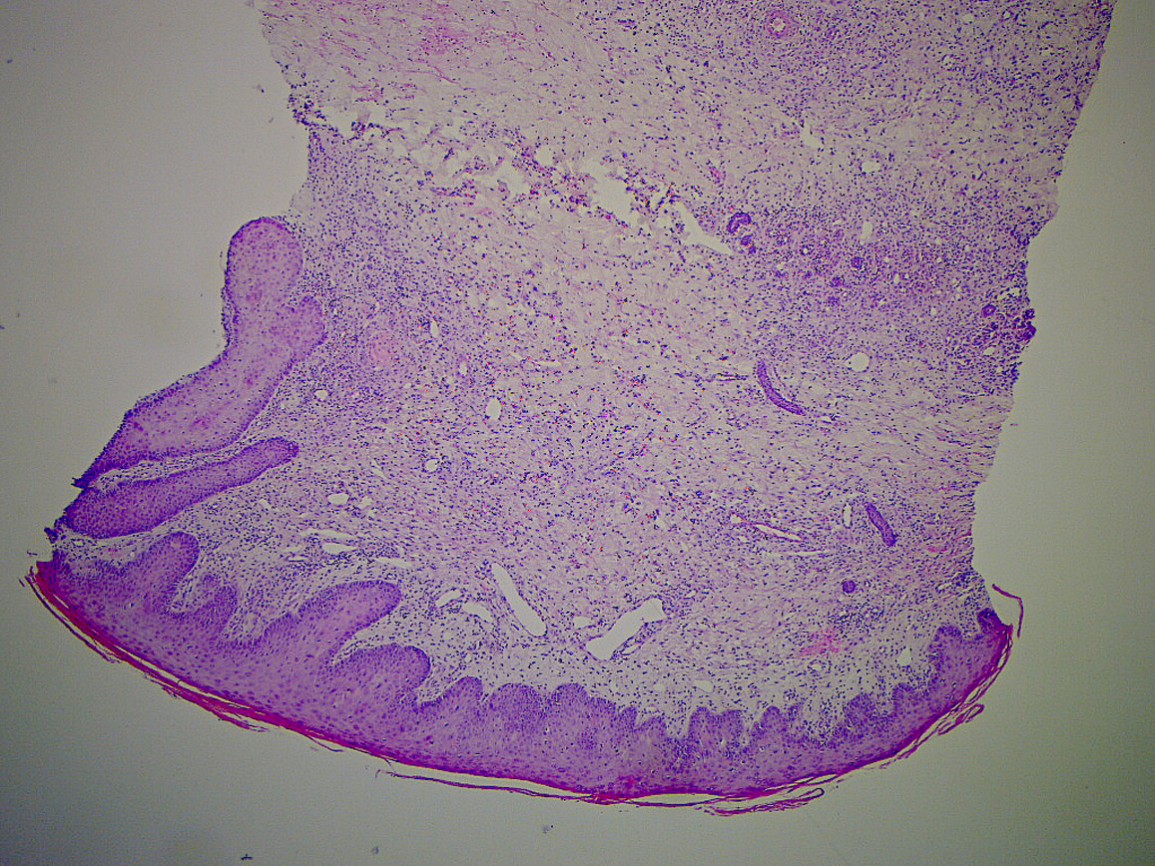
Numerous dilated vessels with moderate dermal inflammatory infiltrate

**FIGURE 2:**
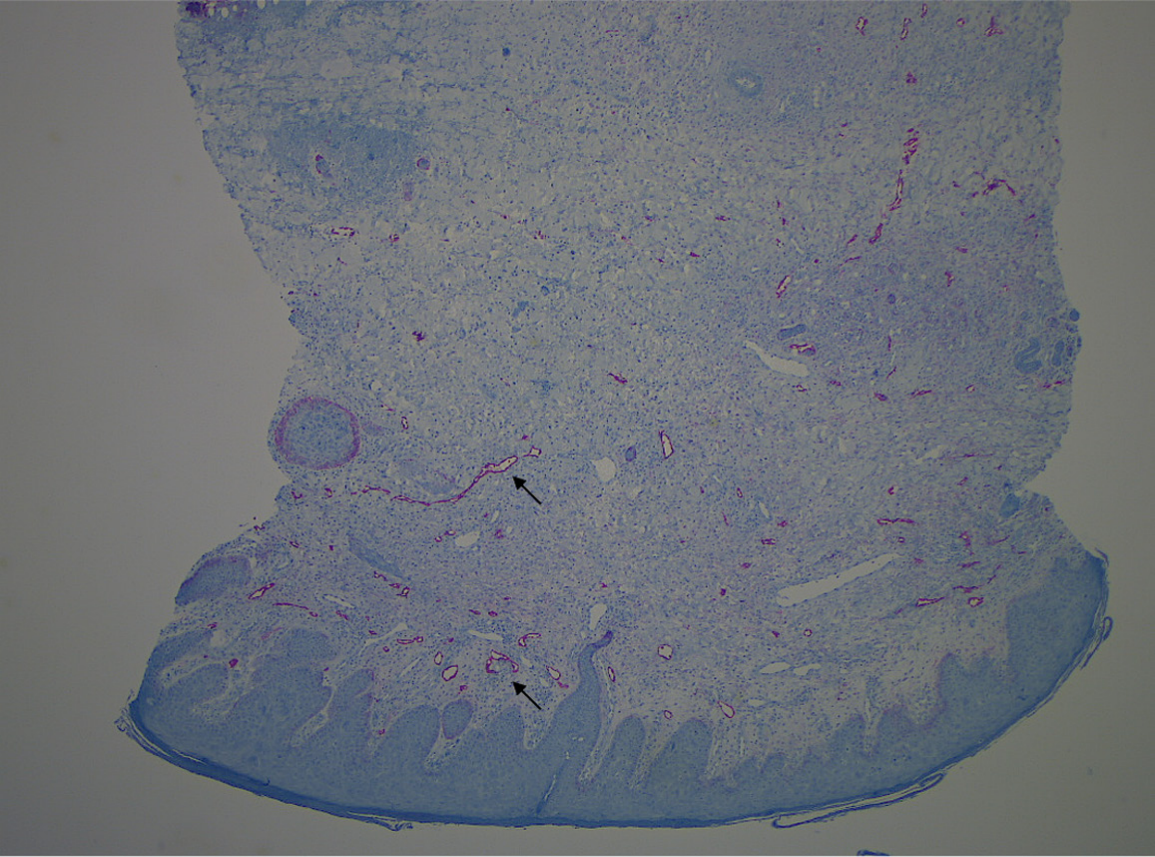
Podoplanin staining with monoclonal antibody D2‐40 revealing numerous lymphatics (black arrows).

**FIGURE 3:**
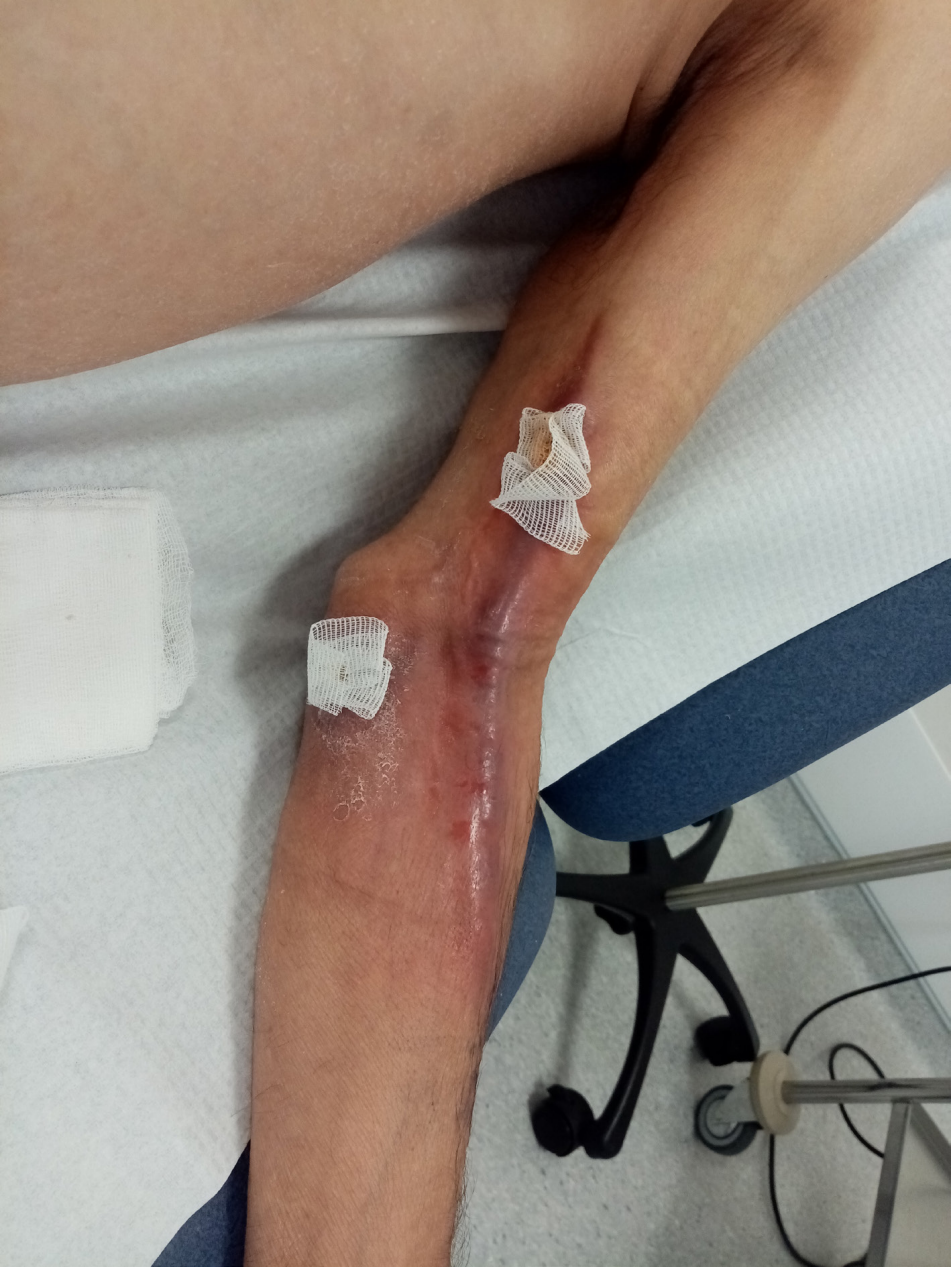
Biopsied skin area on a lymphangitic pathway with an indurated erythematous cord upstream

Lymphatics lack platelets and have high levels of anti‐thrombin, yet they contain all of the coagulation proteins.^1^, ^2^ We assume here that biopsy‐related endothelial alterations and chronic lymph flow obstruction in a pro‐coagulant inflammatory context entailed the lymph thrombosis.^3^, ^4^, ^5^

## AUTHOR CONTRIBUTIONS

Rémy Hamdan conceptualized and designed the study. Rémy Hamdan, Ilham Abejiou, Nicolas Briche, and Ingrid Lafon managed patients. Rémy Hamdan and Lounes Djerroudi interpreted the data. Rémy Hamdan and Nicolas Briche contributed to data acquisition. Rémy Hamdan and Vanessa Gasmi written the manuscript. All authors have read and approved the manuscript.

## FUNDING INFORMATION

None.

## CONFLICT OF INTEREST

None declared.

## CONSENT

Patient consent has been signed and collected in accordance with the journal's patient consent policy.

## Data Availability

Data sharing was not applicable to this article as no datasets were generated or analyzed during the current study.
